# Supplementary material for: Development of a Diagnostic Microfluidic Chip for SARS-CoV-2 Detection in Saliva and Nasopharyngeal Samples
Source: Viruses. 2024 Jul 25;16(8):1190. doi: 10.3390/v16081190 (PMC11360425; doi:10.3390/v16081190)
Supplement: Supplementary file 1 [file viruses-16-01190-s001.zip › viruses-3033097-Supplementary.pdf]

Supplementary information

Development of a diagnostic microfluidic chip for SARS-CoV-2 detection in saliva and nasopharyngeal samples

Sandhya Sharma[a,b], Massimo Caputi[c], Waseem Asghar [a,b,d,*]

[a]Department of Electrical Engineering and Computer Science, Florida Atlantic University, Boca Raton, FL 33431, USA.

[b]Asghar-Lab, Micro and Nanotechnology in Medicine, College of Engineering and Computer Science, Boca Raton, FL 33431, USA.

[c]Charles E. Schmidt College of Medicine, Florida Atlantic University, Boca Raton, FL 33431, USA

[d]Department of Biological Sciences (Courtesy Appointment), Florida Atlantic University, Boca Raton, FL 33431, USA.

* Corresponding Author: Waseem Asghar, Email: wasghar@fau.edu

GENBANK ACCESSION NUMBER	Country	Collection date	Source
LR757997.1	China:Wuhan	12/31/2019	-
MT259238	USA: WA	3/16/2020	-
MT259259	USA: WA	3/16/2020	-
MT198651	Spain: Valencia	3/4/2020	Nasopharyngeal aspirate
MT263409	USA: CT	3/16/2020	-
MT263389	USA: WA	3/16/2020	-
MT259255	USA: WA	3/16/2020	-
MT259270	USA: WA	3/16/2020	-
MT256924	Colombia: Antioquia	3/11/2020	-
MT259272	USA: WA	3/13/2020	-
MT263393	USA: WA	3/14/2020	-
MT233520	Spain: Valencia	2/26/2020	Naso-pharyngeal exudate
MT263407	USA: CT	3/14/2020	-
MT233521	Spain: Valencia	2/27/2020	Nasopharyngeal aspirate
MT246465	USA: WA	3/13/2020	-
MT263460	USA: IL	3/12/2020	-
MT198653	Spain: Valencia	3/8/2020	Nasopharyngeal aspirate
MT259279	USA: WA	3/15/2020	-
MT246483	USA: WA	3/15/2020	-
MT263466	USA: WA	3/16/2020	-
MT259283	USA: WA	3/15/2020	-
MT259242	USA: CT	3/14/2020	-
MT263453	USA: WA	3/24/2020	-
MT259284	USA: WA	3/15/2020	-
MT233522	Spain: Valencia	3/2/2020	Naso-pharyngeal exudate
MT246463	USA: WA	3/13/2020	-
MT246458	USA: WA	3/12/2020	-
MT263385	USA: WA	3/23/2020	-
MT263401	USA: WA	3/15/2020	-
MT259265	USA: WA	3/13/2020	-

MT263451	USA: WA	3/16/2020	-
MT259250	USA: WA	3/14/2020	-
MT259258	USA: MN	3/13/2020	-
MT263428	USA: IL	3/13/2020	-
MT259243	USA: WA	3/13/2020	-
MT259235	USA: WA	3/13/2020	-
MT259240	USA: WA	3/16/2020	-
MT259239	USA: WA	3/16/2020	-
MT263394	USA: WA	3/14/2020	-
MT263390	USA: WA	3/24/2020	-
MT263397	USA: WA	3/24/2020	-
MT163721	USA: WA	3/1/2020	-
MT263462	USA: WA	3/23/2020	-
MT263434	USA: WA	3/24/2020	-
MT233523	Spain: Valencia	3/4/2020	Naso-pharyngeal exudate
MT233519	Spain: Valencia	2/27/2020	Naso-pharyngeal exudate
MT198652	Spain: Valencia	3/5/2020	Tracheal aspirate
MT188339	USA: MN	3/9/2020	-
MT253704	China: Zhejiang, Hangzhou	1/25/2020	Sputum
MT253696	China: Zhejiang, Hangzhou	1/23/2020	Sputum
MT253697	China: Zhejiang, Hangzhou	1/23/2020	Sputum
MT253698	China: Zhejiang, Hangzhou	1/24/2020	Sputum
MT253699	China: Zhejiang, Hangzhou	1/24/2020	Sputum
MT253700	China: Zhejiang, Hangzhou	1/25/2020	Sputum
MT253701	China: Zhejiang, Hangzhou	1/21/2020	Sputum
MT253702	China: Zhejiang, Hangzhou	1/21/2020	Sputum

MT253703	China: Zhejiang, Hangzhou	1/25/2020	Sputum
MT253705	China: Zhejiang, Hangzhou	1/22/2020	Sputum
MT253707	China: Zhejiang, Hangzhou	1/25/2020	Sputum
MT253708	China: Zhejiang, Hangzhou	1/21/2020	Sputum
MT253709	China: Zhejiang, Hangzhou	1/21/2020	Sputum
MT253710	China: Zhejiang, Hangzhou	1/21/2020	Sputum
MT253706	China: Zhejiang, Hangzhou	1/22/2020	Sputum
MT259266	USA: WA	3/13/2020	-
MT263455	USA: WA	3/24/2020	-
MT246485	USA: WA	3/15/2020	-
MT263388	USA: WA	3/16/2020	-
MT259247	USA: WA	3/16/2020	-
MT259287	USA: WA	3/15/2020	-
MT259280	USA: WA	3/15/2020	-
MT259274	USA: WA	3/14/2020	-
MT163720	USA: WA	3/1/2020	-
MT263456	USA: WA	3/24/2020	-
MT259262	USA: WA	3/14/2020	-
MT259241	USA: WA	3/16/2020	-

Table S1. Detailed information of the sequences obtained from the National Center for Biotechnology Information (NCBI) that were used for primer design.

Paper Reference	Sample used	LOD	Limitation/Comments
1	Saliva	5×10^3 copies/mL	External equipment such as smartphones is required to analyze the data.<br>Set-up can process only one sample at a time.
2	Saliva	1×10^4 copies/mL	Detection time is ~2 h.<br>The sample is manually processed.<br>A smartphone is required to record the data.
3	Saliva	5×10^4 genomes/ mL	30 minutes of heat-inactivated treatment is required at 95°C followed by the addition of a TE buffer. Therefore, a trained lab technician is required to handle the testing workflow.
4	Saliva	35×10^4 viral particles per mL	Smartphones are required to analyze the data.<br>The current chip design does not always create an airtight seal. This allows for the sample to flow which can interfere with the true Brownian motion of particles and in turn yield higher diffusion coefficients with higher viscosities than water. After lyophilization, the contact angle of liquid in the chip changes, inducing greater susceptibility to flow.
5	Saliva	2×10^5 copies/mL	External heating devices are required for assay operations.
6	Saliva	500 to 2.5×10^3 copies/m	Manual sample processing is required for viral detection.<br>After the manual sample processing, result wait time is 1 hr.<br>Expensive equipment such as customized heater for heating and smart

			phone is required to anlayze the results
7	Saliva	1.2×10^3 copies/ml	Sample inactivation and reaction take place at 2 different temperatures and chambers therefore human interference is needed.<br>Reaction proceed time is 60 to 120 min.<br>External equipment is required to measure the fluorescent by an expensive BioTek NEO HTS plate reader (BioTek Instruments
8	Saliva	~10^2 viral genomes per reaction	Pre-heat treatment steps are required before saliva processing.<br>The testing was performed using the expensive RT-PCR system in sophisticated lab settings, which is inappropriate for POC settings.
9	Saliva	12 copies per reaction	The study was conducted on a small set of COVID-19 patients.<br>The study presents the most sensitive primers mix analysis on contrived samples using PCR Box UVC/T‑AR in the laboratory. The assay is yet to be developed for POC settings.
11	Saliva/nasopharyngeal samples	20 virions per reaction for nasopharyngeal samples and 93 virions per reaction for saliva samples	Multiple equipment and consumables are needed to run the RT-LAMP assay including saliva collection and lysis tubes, a heat block, pipettes, mineral oil, and a benchtop fluorometer.<br>Pre-heat treatment steps are required before the sample is added to RT-LAMP reagents by trained personnel.<br>Expensive equipment is required such as a heat block and benchtop fluorometer.
12	Saliva	1.5×10^3 copies/mL	Multiple steps for saliva processing are required such as buffer addition for sample dilution, proteinase K addition, and heat-inactivation incubation. To ensure accurate processing of the sample, a trained technician is required

Table S2. Summary of the existing saliva-based rapid molecular tests illustrating the comparison of starting sample type, and limit of detection (LOD) along with the limitations.

Chambers	Reagents	Vol. of reagents	Cost
Inlet chamber	Lysis/Binding buffer	300 μL	$1
	Proteinase K	20 μL	
	Iso-propanol	30 μL	
	Dyna magnetic beads	15 μL	
Wash chamber 1	Washing buffer 1	45 μL	$0.25
Wash chamber 2	Washing buffer 2	45 μL	$0.25
Amplification chamber	LavaLAMP MasterMix	25 μL	$1
	SARS-CoV-2 RT-LAMP primers	5 μL	
	Elution buffer	19 μL	
	MgSO4	2.5 μL	
	SYBR green 1 dye	1 μL	
Oil chambers	Mineral oil (14.50 mPa.s at 25 ℃)	150 μL	$0.50
-	Poly(methyl methacrylate) (PMMA)	-	$0.01
-	Double Sided Adhesive (DSA)	-	$0.01

Table S3. The approximate cost of the reagents and material used for the Microfluidic chip

	Elements	Cost
1	Arduino Uno R3 Microcontroller	$14.00
2	Zip ties	$0.15
3	Screws	$0.50
4	Aluminium rails	$1.50
5	Neodymium Disc Magnets N48	$6.00
6	Surface heater	$14.10
7	Sensor	$12.99

Table S4. List of 3-D printed platform elements and cost

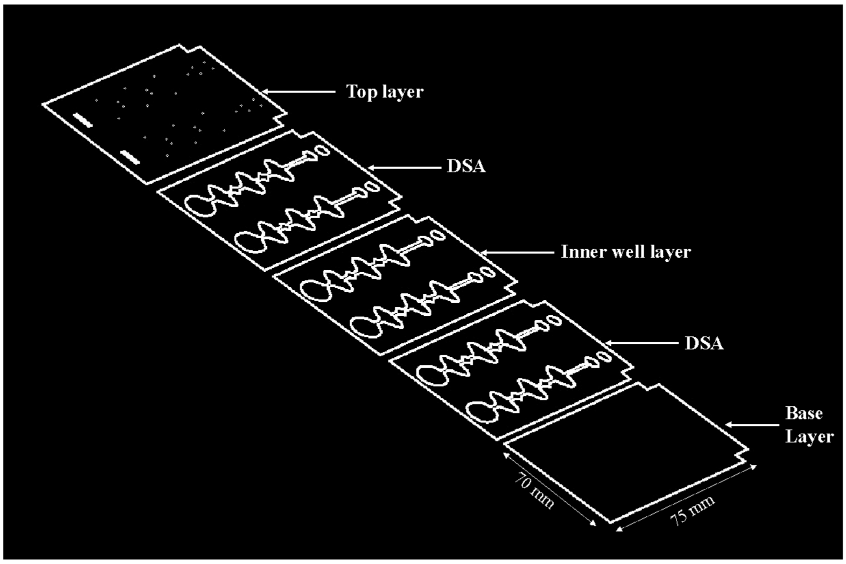

Figure S1. Microfluidic chip fabrication. The microfluidic chip consists of 3 distinct acrylic plastic (PMMA) layers attached using double-sided adhesive (DSA).

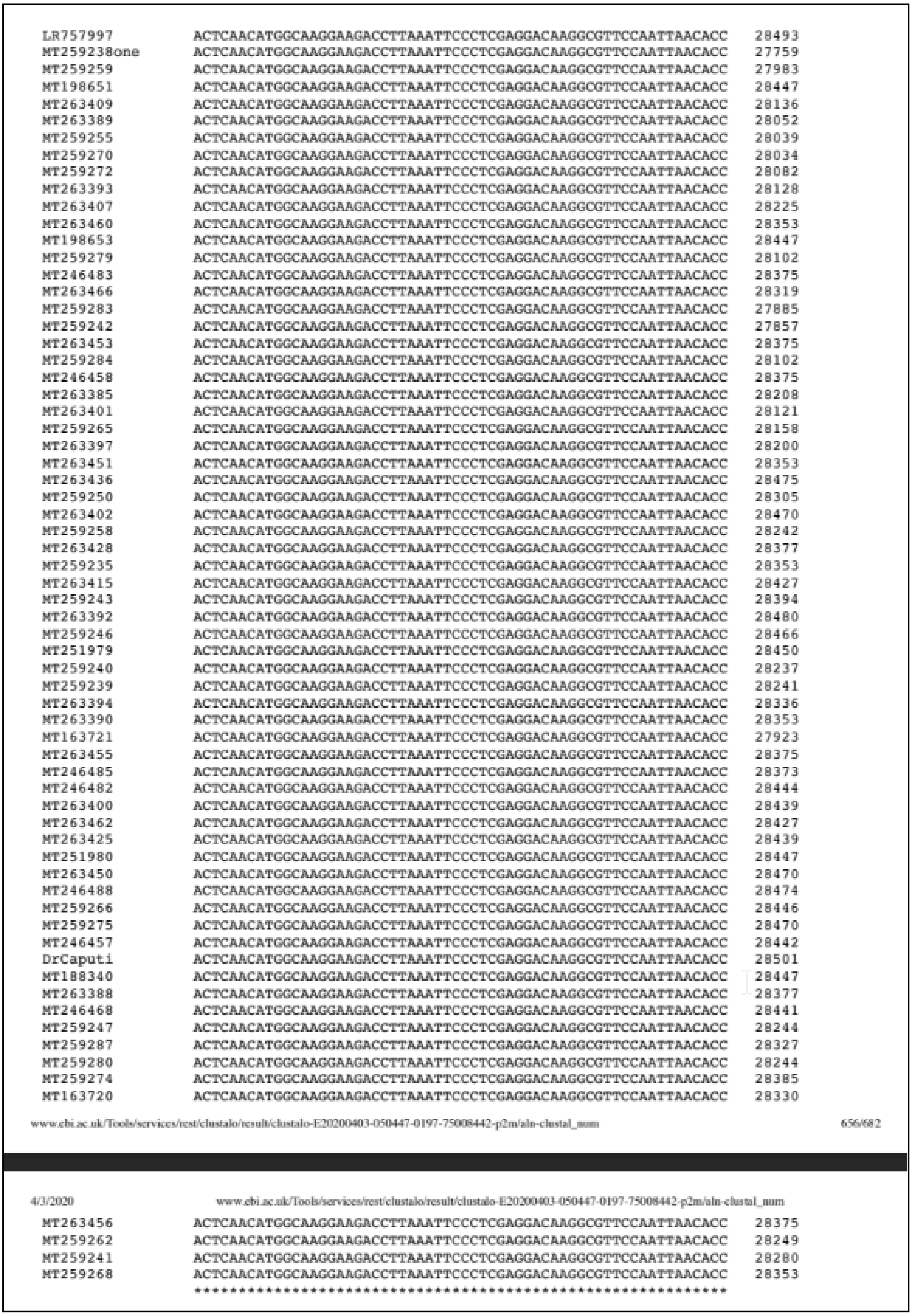

```
LR757997       ACTCAACATGGCAAGGAAGACCTTAAATTCCCTCGAGGACAAGGCGTTCCAATTAACACC   28493
MT259238one    ACTCAACATGGCAAGGAAGACCTTAAATTCCCTCGAGGACAAGGCGTTCCAATTAACACC   27759
MT259259       ACTCAACATGGCAAGGAAGACCTTAAATTCCCTCGAGGACAAGGCGTTCCAATTAACACC   27983
MT198651       ACTCAACATGGCAAGGAAGACCTTAAATTCCCTCGAGGACAAGGCGTTCCAATTAACACC   28447
MT263409       ACTCAACATGGCAAGGAAGACCTTAAATTCCCTCGAGGACAAGGCGTTCCAATTAACACC   28136
MT263389       ACTCAACATGGCAAGGAAGACCTTAAATTCCCTCGAGGACAAGGCGTTCCAATTAACACC   28052
MT259255       ACTCAACATGGCAAGGAAGACCTTAAATTCCCTCGAGGACAAGGCGTTCCAATTAACACC   28039
MT259270       ACTCAACATGGCAAGGAAGACCTTAAATTCCCTCGAGGACAAGGCGTTCCAATTAACACC   28034
MT259272       ACTCAACATGGCAAGGAAGACCTTAAATTCCCTCGAGGACAAGGCGTTCCAATTAACACC   28082
MT263393       ACTCAACATGGCAAGGAAGACCTTAAATTCCCTCGAGGACAAGGCGTTCCAATTAACACC   28128
MT263407       ACTCAACATGGCAAGGAAGACCTTAAATTCCCTCGAGGACAAGGCGTTCCAATTAACACC   28225
MT263460       ACTCAACATGGCAAGGAAGACCTTAAATTCCCTCGAGGACAAGGCGTTCCAATTAACACC   28353
MT198653       ACTCAACATGGCAAGGAAGACCTTAAATTCCCTCGAGGACAAGGCGTTCCAATTAACACC   28447
MT259279       ACTCAACATGGCAAGGAAGACCTTAAATTCCCTCGAGGACAAGGCGTTCCAATTAACACC   28102
MT246483       ACTCAACATGGCAAGGAAGACCTTAAATTCCCTCGAGGACAAGGCGTTCCAATTAACACC   28375
MT263466       ACTCAACATGGCAAGGAAGACCTTAAATTCCCTCGAGGACAAGGCGTTCCAATTAACACC   28319
MT259283       ACTCAACATGGCAAGGAAGACCTTAAATTCCCTCGAGGACAAGGCGTTCCAATTAACACC   27885
MT259242       ACTCAACATGGCAAGGAAGACCTTAAATTCCCTCGAGGACAAGGCGTTCCAATTAACACC   27857
MT263453       ACTCAACATGGCAAGGAAGACCTTAAATTCCCTCGAGGACAAGGCGTTCCAATTAACACC   28375
MT259284       ACTCAACATGGCAAGGAAGACCTTAAATTCCCTCGAGGACAAGGCGTTCCAATTAACACC   28102
MT246458       ACTCAACATGGCAAGGAAGACCTTAAATTCCCTCGAGGACAAGGCGTTCCAATTAACACC   28375
MT263385       ACTCAACATGGCAAGGAAGACCTTAAATTCCCTCGAGGACAAGGCGTTCCAATTAACACC   28208
MT263401       ACTCAACATGGCAAGGAAGACCTTAAATTCCCTCGAGGACAAGGCGTTCCAATTAACACC   28121
MT259265       ACTCAACATGGCAAGGAAGACCTTAAATTCCCTCGAGGACAAGGCGTTCCAATTAACACC   28158
MT263397       ACTCAACATGGCAAGGAAGACCTTAAATTCCCTCGAGGACAAGGCGTTCCAATTAACACC   28200
MT263451       ACTCAACATGGCAAGGAAGACCTTAAATTCCCTCGAGGACAAGGCGTTCCAATTAACACC   28353
MT263436       ACTCAACATGGCAAGGAAGACCTTAAATTCCCTCGAGGACAAGGCGTTCCAATTAACACC   28475
MT259250       ACTCAACATGGCAAGGAAGACCTTAAATTCCCTCGAGGACAAGGCGTTCCAATTAACACC   28305
MT263402       ACTCAACATGGCAAGGAAGACCTTAAATTCCCTCGAGGACAAGGCGTTCCAATTAACACC   28470
MT259258       ACTCAACATGGCAAGGAAGACCTTAAATTCCCTCGAGGACAAGGCGTTCCAATTAACACC   28242
MT263428       ACTCAACATGGCAAGGAAGACCTTAAATTCCCTCGAGGACAAGGCGTTCCAATTAACACC   28377
MT259235       ACTCAACATGGCAAGGAAGACCTTAAATTCCCTCGAGGACAAGGCGTTCCAATTAACACC   28353
MT263415       ACTCAACATGGCAAGGAAGACCTTAAATTCCCTCGAGGACAAGGCGTTCCAATTAACACC   28427
MT259243       ACTCAACATGGCAAGGAAGACCTTAAATTCCCTCGAGGACAAGGCGTTCCAATTAACACC   28394
MT263392       ACTCAACATGGCAAGGAAGACCTTAAATTCCCTCGAGGACAAGGCGTTCCAATTAACACC   28480
MT259246       ACTCAACATGGCAAGGAAGACCTTAAATTCCCTCGAGGACAAGGCGTTCCAATTAACACC   28466
MT251979       ACTCAACATGGCAAGGAAGACCTTAAATTCCCTCGAGGACAAGGCGTTCCAATTAACACC   28450
MT259240       ACTCAACATGGCAAGGAAGACCTTAAATTCCCTCGAGGACAAGGCGTTCCAATTAACACC   28237
MT259239       ACTCAACATGGCAAGGAAGACCTTAAATTCCCTCGAGGACAAGGCGTTCCAATTAACACC   28241
MT263394       ACTCAACATGGCAAGGAAGACCTTAAATTCCCTCGAGGACAAGGCGTTCCAATTAACACC   28336
MT263390       ACTCAACATGGCAAGGAAGACCTTAAATTCCCTCGAGGACAAGGCGTTCCAATTAACACC   28353
MT163721       ACTCAACATGGCAAGGAAGACCTTAAATTCCCTCGAGGACAAGGCGTTCCAATTAACACC   27923
MT263455       ACTCAACATGGCAAGGAAGACCTTAAATTCCCTCGAGGACAAGGCGTTCCAATTAACACC   28375
MT246485       ACTCAACATGGCAAGGAAGACCTTAAATTCCCTCGAGGACAAGGCGTTCCAATTAACACC   28373
MT246482       ACTCAACATGGCAAGGAAGACCTTAAATTCCCTCGAGGACAAGGCGTTCCAATTAACACC   28444
MT263400       ACTCAACATGGCAAGGAAGACCTTAAATTCCCTCGAGGACAAGGCGTTCCAATTAACACC   28439
MT263462       ACTCAACATGGCAAGGAAGACCTTAAATTCCCTCGAGGACAAGGCGTTCCAATTAACACC   28427
MT263425       ACTCAACATGGCAAGGAAGACCTTAAATTCCCTCGAGGACAAGGCGTTCCAATTAACACC   28439
MT251980       ACTCAACATGGCAAGGAAGACCTTAAATTCCCTCGAGGACAAGGCGTTCCAATTAACACC   28447
MT263450       ACTCAACATGGCAAGGAAGACCTTAAATTCCCTCGAGGACAAGGCGTTCCAATTAACACC   28470
MT246488       ACTCAACATGGCAAGGAAGACCTTAAATTCCCTCGAGGACAAGGCGTTCCAATTAACACC   28474
MT259266       ACTCAACATGGCAAGGAAGACCTTAAATTCCCTCGAGGACAAGGCGTTCCAATTAACACC   28446
MT259275       ACTCAACATGGCAAGGAAGACCTTAAATTCCCTCGAGGACAAGGCGTTCCAATTAACACC   28470
MT246457       ACTCAACATGGCAAGGAAGACCTTAAATTCCCTCGAGGACAAGGCGTTCCAATTAACACC   28442
DrCaputi       ACTCAACATGGCAAGGAAGACCTTAAATTCCCTCGAGGACAAGGCGTTCCAATTAACACC   28501
MT188340       ACTCAACATGGCAAGGAAGACCTTAAATTCCCTCGAGGACAAGGCGTTCCAATTAACACC   28447
MT263388       ACTCAACATGGCAAGGAAGACCTTAAATTCCCTCGAGGACAAGGCGTTCCAATTAACACC   28377
MT246468       ACTCAACATGGCAAGGAAGACCTTAAATTCCCTCGAGGACAAGGCGTTCCAATTAACACC   28441
MT259247       ACTCAACATGGCAAGGAAGACCTTAAATTCCCTCGAGGACAAGGCGTTCCAATTAACACC   28244
MT259287       ACTCAACATGGCAAGGAAGACCTTAAATTCCCTCGAGGACAAGGCGTTCCAATTAACACC   28327
MT259280       ACTCAACATGGCAAGGAAGACCTTAAATTCCCTCGAGGACAAGGCGTTCCAATTAACACC   28244
MT259274       ACTCAACATGGCAAGGAAGACCTTAAATTCCCTCGAGGACAAGGCGTTCCAATTAACACC   28385
MT163720       ACTCAACATGGCAAGGAAGACCTTAAATTCCCTCGAGGACAAGGCGTTCCAATTAACACC   28330
```

www.ebi.ac.uk/Tools/services/rest/clustalo/result/clustalo-E20200403-050447-0197-75008442-p2m/aln-clustal_num 656/682

4/3/2020 www.ebi.ac.uk/Tools/services/rest/clustalo/result/clustalo-E20200403-050447-0197-75008442-p2m/aln-clustal_num

```
MT263456       ACTCAACATGGCAAGGAAGACCTTAAATTCCCTCGAGGACAAGGCGTTCCAATTAACACC   28375
MT259262       ACTCAACATGGCAAGGAAGACCTTAAATTCCCTCGAGGACAAGGCGTTCCAATTAACACC   28249
MT259241       ACTCAACATGGCAAGGAAGACCTTAAATTCCCTCGAGGACAAGGCGTTCCAATTAACACC   28280
MT259268       ACTCAACATGGCAAGGAAGACCTTAAATTCCCTCGAGGACAAGGCGTTCCAATTAACACC   28353
               ************************************************************
```

Figure S2a. Snapshot of the sequences aligned in Clustal W, asterisk sign (*) at the bottom shows the conserved nucleotide in all the sequences.

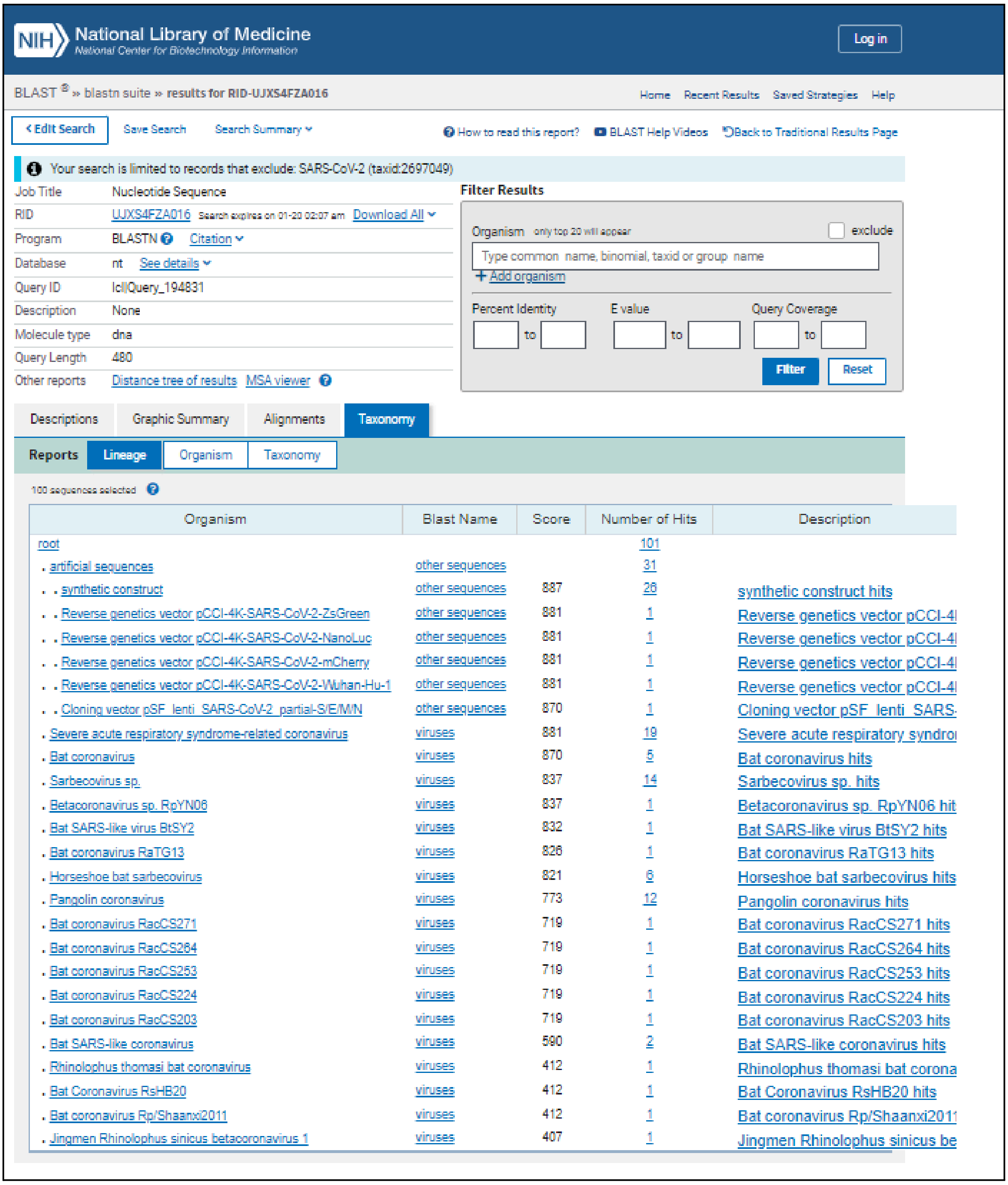

Figure S2b. Snapshot of the NCBI-BLAST Lineage results of the conserved target N-gene sequence after excluding SARS-CoV-2 (taxid:2697049).

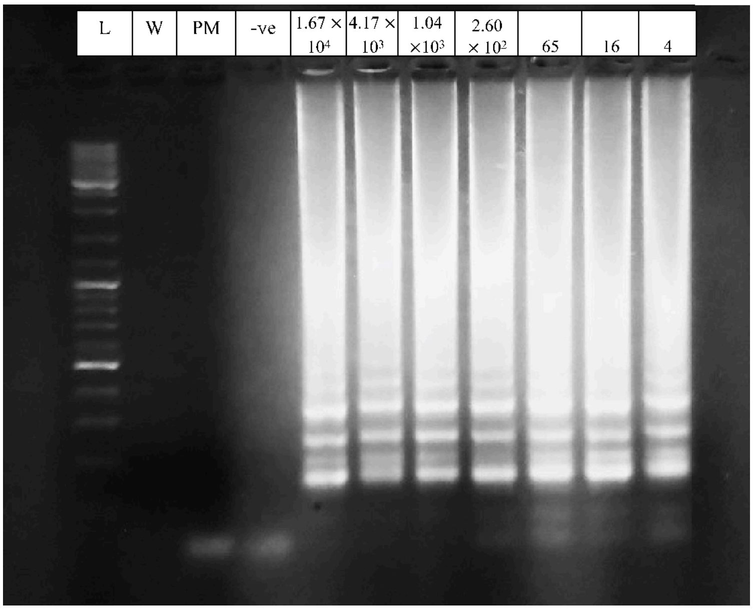

Figure S3. 1.5% gel electrophoresis results stained with Bromophenol blue dye (lane L contains 1 kbp size DNA ladder). Sharp bands are observed in the wells containing the LAMP amplification product of SARS-CoV2 from 10^4 to 4 copies/reaction.

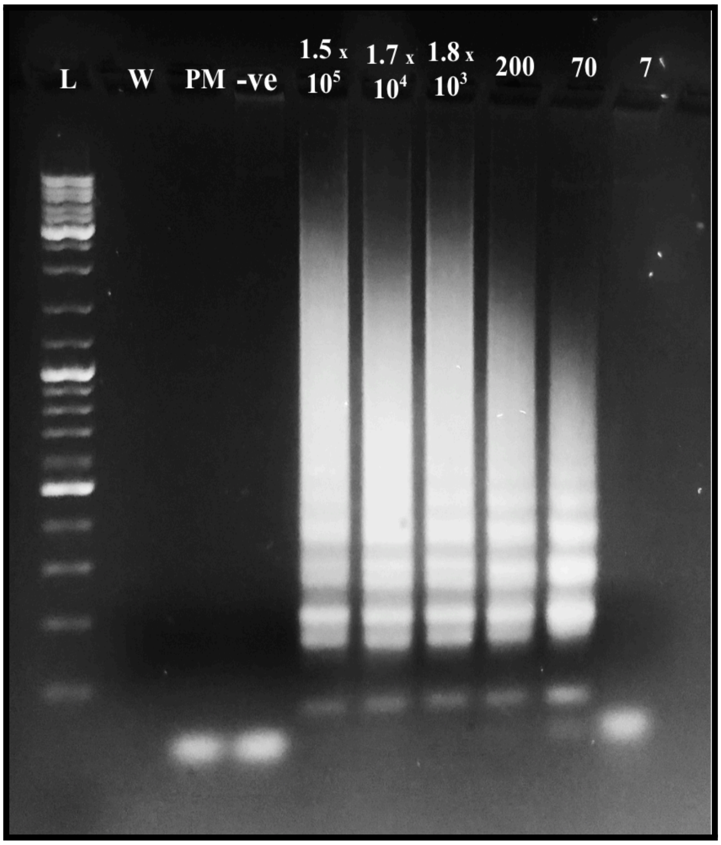

Figure S4. 1.5% gel electrophoresis results stained with Bromophenol blue dye (lane L contains 1 kbp size DNA ladder). Sharp bands in the wells holding the LAMP amplification product of SARS-CoV2 target RNA and relative RNA copies/reaction utilized in each reaction.

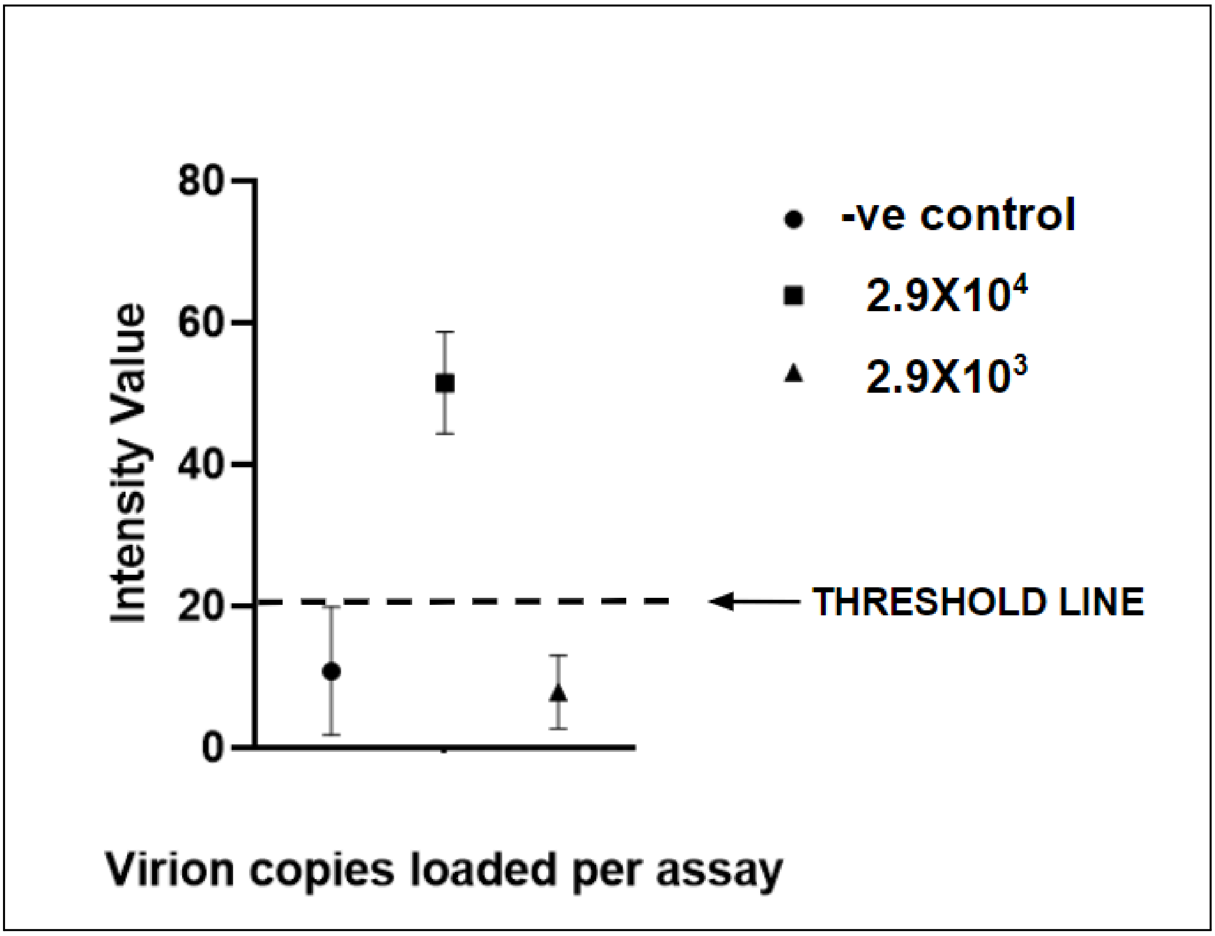

Figure S5. The gray value plot of the on-chip saliva sample results in a saturated reaction measured by converting RGB pixel to average gray intensity using ImageJ (Defined threshold value=20).

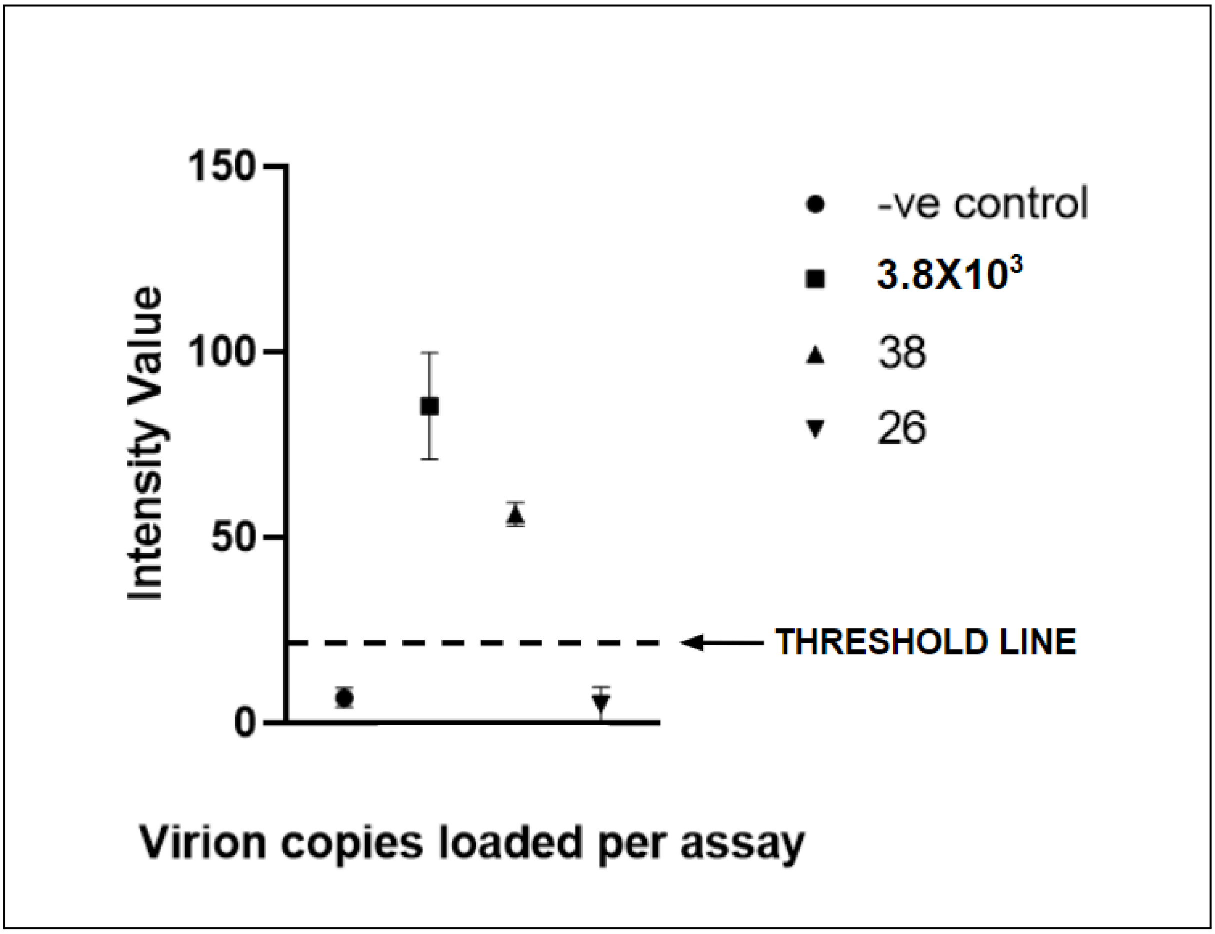

Figure S6. The gray value plot of the on-chip nasopharyngeal sample results in a saturated reaction measured by converting RGB pixel to average gray intensity using ImageJ (Defined threshold value=20).

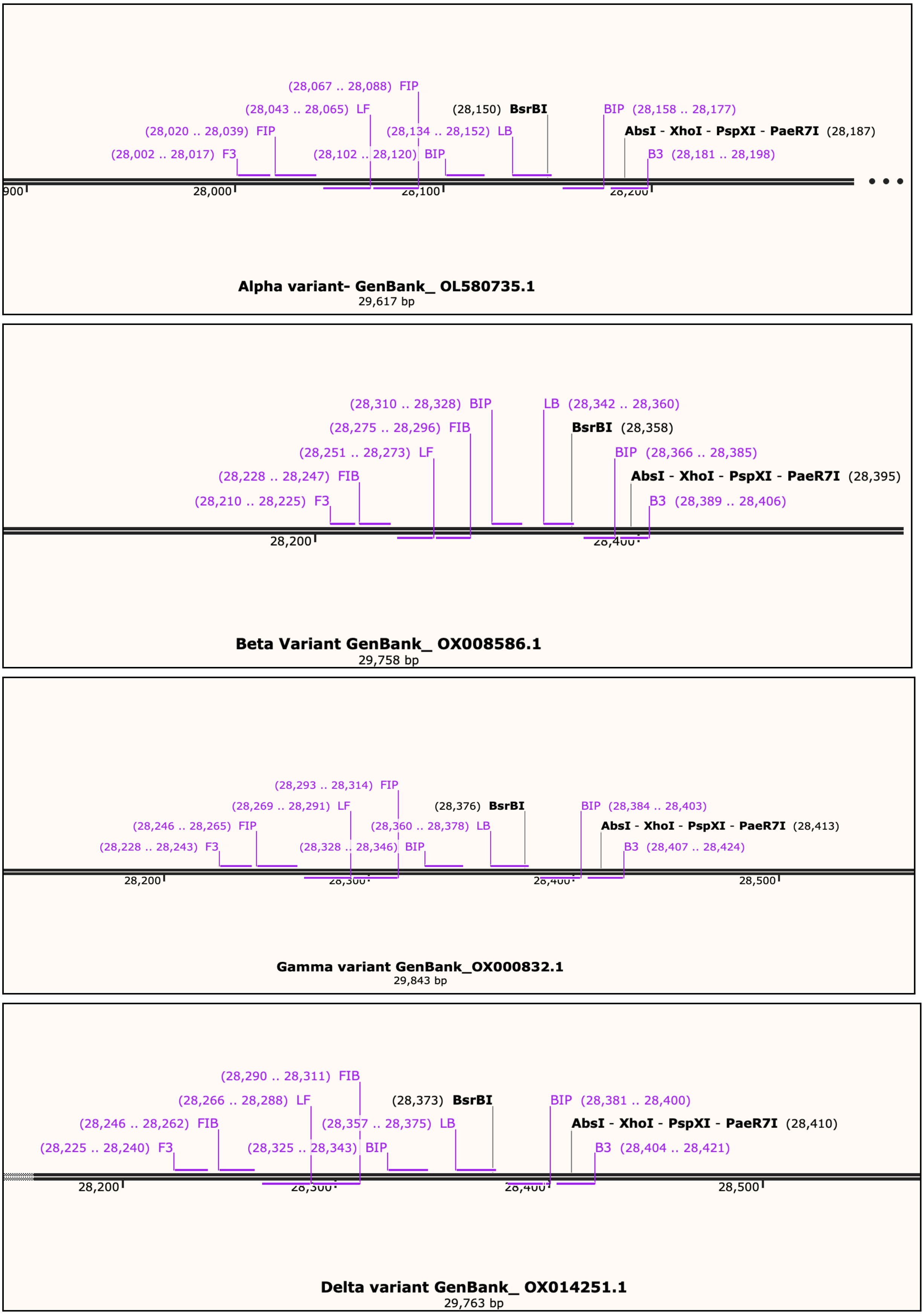
(28,067 .. 28,088) FIP
(28,043 .. 28,065) LF
(28,150) BsrBI
BIP (28,158 .. 28,177)
(28,020 .. 28,039) FIP
(28,134 .. 28,152) LB
AbsI - XhoI - PspXI - PaeR7I (28,187)
(28,002 .. 28,017) F3
(28,102 .. 28,120) BIP
B3 (28,181 .. 28,198)
28,000
28,100
Alpha variant- GenBank_ OL580735.1
29,617 bp
(28,310 .. 28,328) BIP
LB (28,342 .. 28,360)
(28,275 .. 28,296) FIB
BsrBI (28,358)
(28,251 .. 28,273) LF
BIP (28,366 .. 28,385)
(28,228 .. 28,247) FIB
AbsI - XhoI - PspXI - PaeR7I (28,395)
(28,210 .. 28,225) F3
B3 (28,389 .. 28,406)
28,200
Beta Variant GenBank_ OX008586.1
29,758 bp
(28,293 .. 28,314) FIP
(28,269 .. 28,291) LF
(28,376) BsrBI
BIP (28,384 .. 28,403)
(28,246 .. 28,265) FIP
(28,360 .. 28,378) LB
AbsI - XhoI - PspXI - PaeR7I (28,413)
(28,228 .. 28,243) F3
(28,328 .. 28,346) BIP
B3 (28,407 .. 28,424)
28,200
28,500
Gamma variant GenBank_OX000832.1
29,843 bp
(28,290 .. 28,311) FIB
(28,266 .. 28,288) LF
(28,373) BsrBI
BIP (28,381 .. 28,400)
(28,246 .. 28,262) FIB
(28,357 .. 28,375) LB
AbsI - XhoI - PspXI - PaeR7I (28,410)
(28,225 .. 28,240) F3
(28,325 .. 28,343) BIP
B3 (28,404 .. 28,421)
28,200
28,500
Delta variant GenBank_ OX014251.1
29,763 bp

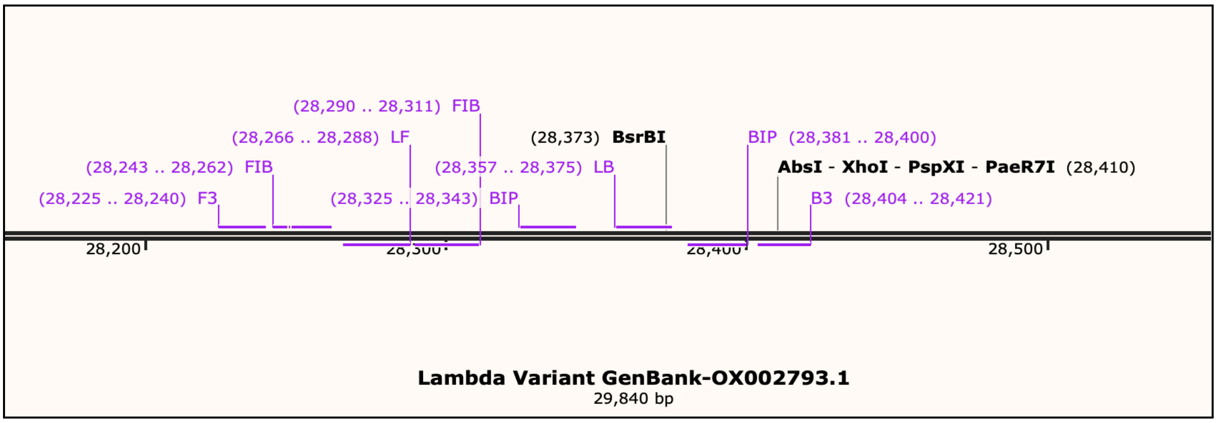

Figure S7. Designed RT-LAMP primer aligned against the Alpha, Beta, Gamma, Delta and Lambda SARS-CoV-2 variants in the SnapGene Viewer software illustrating the conserved primers' annealing sites on the genome.

References

1. Zifan T. *et al.* SLIDE: Saliva-Based SARS-CoV-2 Self-Testing with RT-LAMP in a Mobile Device. *ACS Sens*, *7* **(8)**, 2370-2378 (2022)
2. Jongwon L. *et al.* Microfluidic point-of-care device for detection of early strains and B.1.1.7 variant of SARS-CoV-2 virus. *Lab Chip*, **22**, 1297-1309 (2022)
3. Diaz LM, *et al*. Real-time optical analysis of a colorimetric LAMP assay for SARS-CoV-2 in saliva with a handheld instrument improves accuracy compared with endpoint assessment. *J Biomol Tech.* **32(3)**: 158-171 (2021).
4. Colbert A.J, *et al*. PD-LAMP smartphone detection of SARS-CoV-2 on chip. *Anal Chim Acta*. **22**;1203:339702 (2022).
5. Davidson JL, *et al*. A paper-based colorimetric molecular test for SARS-CoV-2 in saliva. *Biosens Bioelectron X*. **9** (2021)
6. Panpradist N, *et al*. Harmony COVID-19: A ready-to-use kit, low-cost detector, and smartphone app for point-of-care SARS-CoV-2 RNA detection. *Sci Adv.* **17**; 7(51) (2021)
7. Helena de Puig *et al.*, Minimally instrumented SHERLOCK (miSHERLOCK) for CRISPR-based point-of-care diagnosis of SARS-CoV-2 and emerging variants. *Sci. Adv.***7**, 2944 (2021).
8. Lalli MA, *et al.* Rapid and Extraction-Free Detection of SARS-CoV-2 from Saliva by Colorimetric Reverse-Transcription Loop-Mediated Isothermal Amplification. *Clin Chem*. **30**;67(2):415-424 (2021)
9. Monika J., *et al.* Loop-mediated isothermal amplification for the detection of SARS-CoV-2 in saliva. *Microbial Biotech,* **14**; 307-316 (2021)
10. Kundrod K.A., *et al.* Sample-to-answer, extraction-free, real-time RT-LAMP test for SARS-CoV-2 in nasopharyngeal, nasal, and saliva samples: Implications and use for surveillance testing. *PLoS One*. **17**(2) (2022)
11. Yamazaki W., *et al.* Development of a point-of-care test to detect SARS-CoV-2 from saliva which combines a simple RNA extraction method with colorimetric reverse transcription loop-mediated isothermal amplification detection. *J Clin Virol*. **136**:104760 (2021).
12. Huang X., *et al.* Developing RT-LAMP assays for rapid diagnosis of SARS-CoV-2 in saliva. *EBioMedicine*. **75**:103736 (2022).
